# Beyond tumors: uninvolved breast tissue of breast cancer patients with adverse prognoses is enriched for pathogenic *PIK3CA* and *TP53* post-zygotic variants

**DOI:** 10.1186/s12885-026-16086-z

**Published:** 2026-04-30

**Authors:** Maria Andreou, Katarzyna Chojnowska, Natalia Filipowicz, Monika Horbacz, Piotr Madanecki, Katarzyna Duzowska, Urszula Ławrynowicz, Hanna Davies, Bożena Bruhn-Olszewska, Mikołaj Koszyński, Kinga Drężek-Chyła, Maciej Jaśkiewicz, Marcin Jąkalski, Anna Kostecka, Marta Drzewiecka-Kłysz, Magdalena Nowikiewicz, Manuela Las-Jankowska, Dariusz Bała, Jacek Hoffman, Ewa Śrutek, Michał Jankowski, Jerzy Jankau, Diana Hodorowicz-Zaniewska, Joanna Szpor, Łukasz Szylberg, Wojciech Zegarski, Tomasz Nowikiewicz, Patrick G. Buckley, Irene Tiemann-Boege, Jakub Mieczkowski, Magdalena Koczkowska, Jan P. Dumanski, Arkadiusz Piotrowski

**Affiliations:** 1 3P-Medicine Laboratory, Medical University of Gdańsk, Gdańsk, Poland; 2 Department of Biology and Pharmaceutical Botany, Medical University of Gdańsk, Gdańsk, Poland; 3 Department of Immunology, Genetics and Pathology and Science for Life Laboratory, Uppsala University, Uppsala, Sweden; 4 Department of Clinical Breast Cancer and Reconstructive Surgery, Oncology Center-Prof. Franciszek Łukaszczyk Memorial Hospital, Bydgoszcz, Poland; 5 Department of Hepatobiliary and General Surgery, A. Jurasz University Hospital, Bydgoszcz, Poland; 6 Chair of Surgical Oncology, Ludwik Rydygier’s Collegium Medicum, Nicolaus Copernicus University, Bydgoszcz, Toruń, Poland; 7 Department of Clinical Oncology, Oncology Center-Prof. Franciszek Łukaszczyk Memorial Hospital, Bydgoszcz, Poland; 8 Department of Surgical Oncology, Oncology Center-Prof. Franciszek Łukaszczyk Memorial Hospital, Bydgoszcz, Poland; 9 Surgical Oncology, Ludwik Rydygier’s Collegium Medicum, Nicolaus Copernicus University, Bydgoszcz, Toruń, Poland; 10 Department of Tumor Pathology and Pathomorphology, Oncology Center-Prof Franciszek Łukaszczyk Memorial Hospital, Bydgoszcz, Poland; 11 Department of Plastic Surgery, Medical University of Gdańsk, Gdańsk, Poland; 12 1st Department of General Surgery, Breast Unit University Hospital, Jagiellonian University Medical College, Krakow, Poland; 13 Department of Pathomorphology, Jagiellonian University Medical College, Kraków, Poland; 14 Department of Clinical Pathomorphology, Collegium Medicum in Bydgoszcz, Nicolaus Copernicus University, Toruń, Poland; 15 Genseq, Dublin, Ireland; 16 Institute of Biophysics, Johannes Kepler University, Linz, Austria

**Keywords:** Breast cancer, Recurrence, Post-zygotic variants, Unfavorable outcome, Uninvolved mammary gland

## Abstract

**Background:**

Histologically normal mammary tissue from breast cancer patients can harbor significant genetic alterations that could precede visible tumor development and influence disease progression.

**Methods:**

Whole-exome sequencing was performed on 408 samples from 77 breast cancer patients with poor prognosis, 49 patients recruited without prognosis-based selection, and 15 individuals undergoing non-cancer-related mammoplasty. Paired primary tumor and histologically normal mammary gland tissues were analyzed. Variant classification adhered to strict filtering criteria, incorporating allele frequency thresholds, multiple annotation databases, and in silico prediction tools. Duplex sequencing was employed to detect and confirm pathogenic *PIK3CA* and *TP53* variants in normal mammary tissue samples from 11 breast cancer patients with unfavorable prognosis. Statistical analyses included hypergeometric testing, Kaplan–Meier survival analysis, and Cox proportional hazards modeling.

**Results:**

Post-zygotic pathogenic variants in cancer-associated genes were significantly more prevalent in normal mammary tissue of poor-prognosis patients (29%) than in unselected patients (12.5%) (*p* = 0.0008578). Variant presence and per-individual burden were similar across age-matched cohorts and intrinsic subtypes, indicating that subtype composition, germline predisposition and age do not account for the excess UM variant load in BCAP. Truncating variants were exclusive to poor-prognosis cases. Frequently altered genes included *AKT1*,* PIK3CA*,* PTEN*,* TBX3*, and *TP53*, with *TP53* variants detected only in patients with adverse outcomes. Duplex sequencing confirmed the presence of low-frequency variants (as low as 1.34%) in regions of histologically normal breast tissue from patients with a poor prognosis. Notably, nearly one-quarter of all identified cases (24%, 12/49) harbored pathogenic variants in normal tissue absent from corresponding primary tumors, suggesting that at least some mosaic clones in uninvolved mammary tissue represent independent evolutionary events rather than residual tumor cells.

**Conclusions:**

Post-zygotic pathogenic variants are frequent in histologically normal mammary tissue from breast cancer patients, including alterations in key cancer-associated genes. These findings indicate that mosaic clonal changes outside the tumor are more common than previously appreciated and warrant further investigation. Assessing such variants in non-tumorous tissue may, in the future, help refine approaches to breast cancer risk evaluation and management.

**Supplementary Information:**

The online version contains supplementary material available at 10.1186/s12885-026-16086-z.

## Background

Breast cancer accounts for 12.5% of global annual cancer diagnoses, with the incidence rate increasing by 0.5% annually in recent years [1, 2]. Despite an overall 42% reduction between 1989 and 2021, primarily attributed to increased awareness and early detection, breast cancer still constitutes one of the leading causes of death among women [3]. Notably, stage I, low-risk breast cancer cases still present a 15–20% chance of recurrence even two decades after the initial diagnosis [4]. While 5–10% of breast cancer cases are hereditary, with 25–30% of heritable breast cancer risk attributed to pathogenic variants in genes of high and moderate penetrance [5], the majority of cases are considered sporadic [6, 7].

Research has recently focused on the normal mammary gland within the affected breast for early detection of tumor formation at the molecular level, preceding changes on imaging or palpative screens [8]. Concurrently, breast-conserving surgery (BCS), which aims to remove the tumor and preserve the remaining healthy breast tissue, stands as the preferred approach [9, 10]. However, mammary gland tissue from breast cancer patients, although appearing normal, has been found to harbor significant genomic and transcriptomic alterations [11–14]. In particular, non-tumorous tissue from patients undergoing BCS shows clearly pathogenic low-level post-zygotic alterations in the *PIK3CA* and *TP53* genes, raising questions regarding its oncogenic potential [15, 16].

Nevertheless, the association between post-zygotic alterations in ostensibly normal mammary gland tissue of breast cancer patients and their clinical utility remains unclear. Hence, we screened paired uninvolved mammary gland (UM) and primary tumor (PT) samples of reportedly sporadic breast cancer patients with adverse outcomes within 10 years post-original surgery for the presence of post-zygotic alterations. We compared our findings with a second breast cancer cohort of reportedly sporadic patients recruited without specific prognosis criteria. Additionally, normal mammary gland samples from individuals with no personal or family history of cancer were included as controls (study overview in Additional File 1, Supplementary Fig. 1).

Here, we demonstrate that pathogenic post-zygotic variants in cancer-associated genes are commonly found in histologically normal mammary tissue of breast cancer patients with adverse prognoses. These variants, often also found in corresponding primary tumors, are linked with patient survival, emphasizing the need for molecular screening to improve the clinical management of patients.

## Methods

### Patient recruitment, sample collection, and DNA isolation

We carried out Whole Exome Sequencing (WES) on 408 samples from three distinct groups: two cohorts of female breast cancer patients with differing prognostic outcomes and a control group composed of female individuals who underwent mammoplasty surgery for non-cancer-related reasons. The assignment of patients to the cohorts was determined by their clinical prognoses. The first cohort included 77 reportedly sporadic breast cancer patients with adverse outcomes. All individuals in this group experienced either recurrent disease, such as local recurrence/metastasis to the breast or secondary organs (*n* = 40), developed a second independent tumor (*n* = 18), or both (*n* = 8), and/or succumbed to the disease (*n* = 45) within the proceeding 10 years (*B*reast *C*ancer *A*dverse *P*rognoses cohort, BCAP), (Table 1; Additional File 2:Supplementary Table 1). The second cohort included 49 individuals from the same ethnic population, diagnosed with reportedly sporadic breast cancer but recruited without specific criteria related to prognosis (*B*reast *C*ancer *U*n-*S*elected cohort, BCUS). Within this group, 5 out of 49 patients experienced recurrence, and 3 of them died within 2 years post-surgery; however, the follow-up period for this cohort was considerably shorter compared to the BCAP cohort (Table 1; Additional File 2: Supplementary Table 1). The majority of BCAP and BCUS patients were treated with BCS (*n* = 63 and *n* = 31, respectively) versus mastectomy (*n* = 12 and *n* = 18, respectively) (Additional File 2: Supplementary Table 1) (data missing for 2 BCAP patients). All recruited individuals did not receive neoadjuvant therapy. The control group comprised 15 individuals who underwent reduction mammoplasty surgeries and had no personal or familial history of cancer (*R*eduction *M*ammoplasty cohort, RM). Written informed consent was obtained from all enrolled individuals. The study was approved by the the Bioethical Committee at the Collegium Medicum, Nicolaus Copernicus University in Toruń (approval number KB509/2010) and by the Independent Bioethics Committee for Research at the Medical University of Gdańsk (approval number NKBBN/564/2018 with multiple amendments), recruited and enrolled all donors under informed and written consent, collected, and stored all tissue samples. A total of 415 samples, including PT, UM, blood (BL), or skin (SK) from all three cohorts were collected by the Oncology Centre in Bydgoszcz, Jagiellonian University Hospital in Cracow, and the University Clinical Centre in Gdańsk, with the necessary ethical approvals and written informed consent from participants and deposited in the biobank of our unit at the Medical University of Gdańsk, along with clinical data, including follow-up information (Table 1; Additional File 2: Supplementary Table 1). Distal UM samples (UMD, 1.5–3 cm from PT, median 2.35 cm), available for 7 BCAP patients, were collected and included in the downstream targeted confirmatory analysis; however, they were not initially sequenced. For the RM cohort, sets of UM and BL samples were included. UM samples were located at least 1 cm away from the corresponding PT. All collected samples were frozen at -80 °C. Detailed tissue-collecting protocols were previously described by Filipowicz et al. [17]. All fragments prepared for molecular analysis were histologically evaluated by expert pathologists to identify tumor fragments (PT) and confirm the normal histology of UM and SK samples. DNA isolation from tissue lysates and whole blood was performed as previously described [17].

**Table 1:** Summarized clinicopathological features of breast cancer patients included in the BCAP and BCUS cohorts

	BCAP cohort	BCUS cohort
Number of individuals	77	49
Age (median, range)	62, 23–85	65, 37–84
		*p* value = 0.082
Collected samples	238	147
Primary Tumor, PT	77	49
Uninvolved mammary gland, UM	77	49
Distal fragment of uninvolved mammary gland, UMD	7	-
Reference sample (whole peripheral blood, BL or skin, SK)	77	49
Histology		
Invasive ductal carcinoma, IDC	59	40
Invasive lobular carcinoma, ILC	3	4
IDC - ILC	6	1
other	9	4
Grade		
Grade 1	1 (77)	5 (49)
		*p* value = 2.1 × 10⁻⁵
Grade 2	43 (77)	40 (49)
Grade 3–4	32 (77)	3 (49)
Receptors		
Estrogen, ER (positive / negative / not available)	57 / 20	43 / 5 / 1
ER positive		*p* value = 0.040
Progesterone, PR (positive / negative / not available)	43 / 34	44 / 4 / 1
PR positive		*p* value = 1.7 × 10⁻⁵
HER2 (positive / negative / not available)	16 / 56 / 5	5 / 43 / 1
HER3 positive		*p* value = 0.14
Subtype		
Luminal A	14 (77)	22 (49)
		*p* value = 0.0025
Luminal B	37 (77)	21 (49)
HER-2 enriched	9 (77)	2 (49)
Triple-negative breast cancer, TNBC	11 (77)	1 (49)
No data	6 (77)	3 (49)
Follow-up information		
Recurrence (yes / no)	50 / 27	5 / 44
Second cancer (yes / no)	26 / 51	0 / 49
Death* (yes / no)	45 / 31	3 / 46

**Fig. 1:**
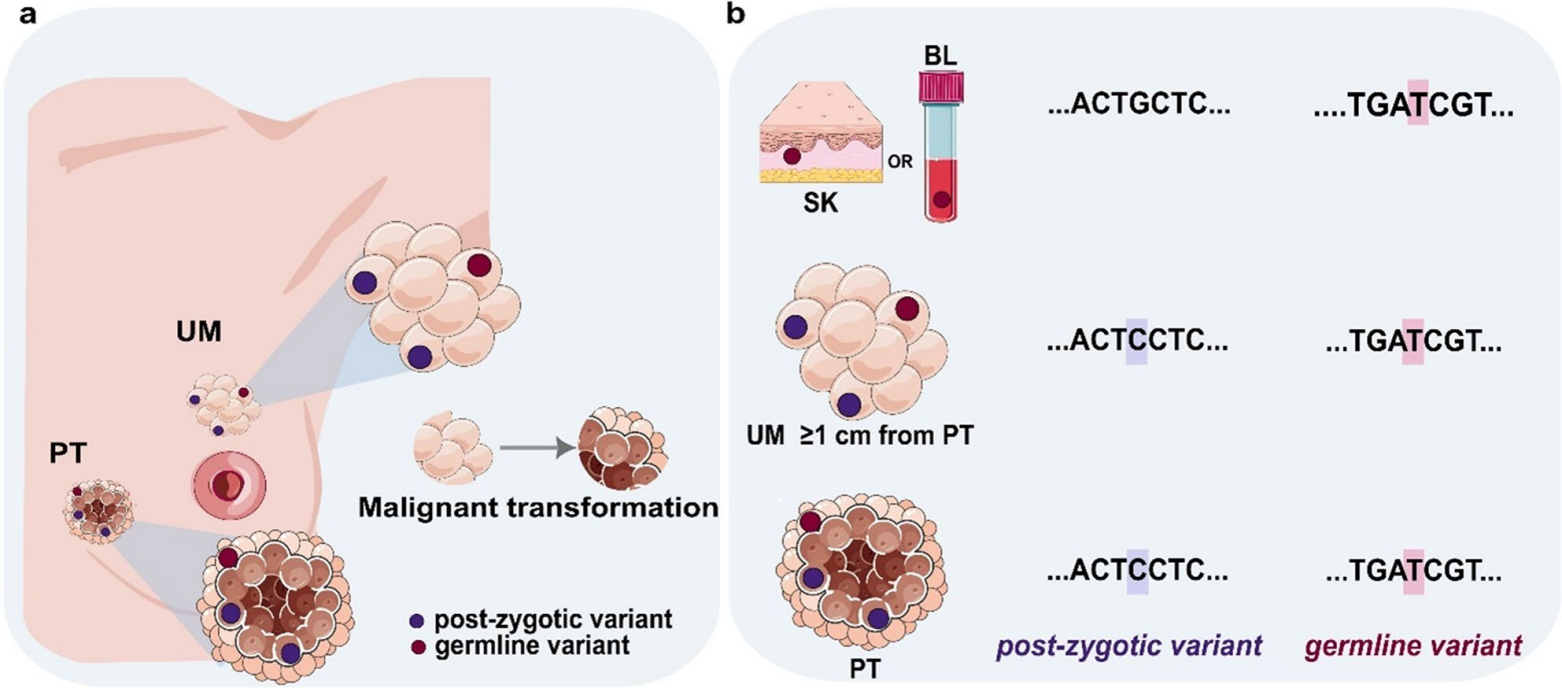
Graphical representation of (**a**) sampling design and (**b**) investigation of post-zygotic and germline variants. (**a) **Matched fresh-frozen uninvolved mammary gland (UM) and primary tumor (PT) samples were collected from all breast cancer patients to identify variants according to study criteria (Methods). UM samples were located at least 1 cm away from the corresponding PT. Control mammary gland samples were obtained from individuals who underwent reduction mammoplasty surgeries and had no history of cancer (Reduction Mammoplasty cohort, RM). (**b) **Matched peripheral blood (BL) or skin (SK) samples were collected for each individual as reference samples to distinguish between post-zygotic and germline variants. Post-zygotic variants were identified as those that were absent from the reference samples

Matched and primary tumor (PT) and uninvolved mammary gland (UM, ≥ 1 cm ) samples were collected from two breast cancer cohorts, i.e., 77 individuals characterized with adverse outcomes (Breast Cancer Adverse Prognoses cohort, BCAP) and 49 individuals recruited without any pre-selection criteria related to prognosis (Breast Cancer Un-Selected cohort, BCUS ). Whole peripheral blood (BL) or skin (SK) samples (if BL was not available) were collected as reference samples to distinguish between post-zygotic and germline variants. Distal UM samples (UMD, 1.5–3 cm from PT, median 2.35 cm), available for 7 BCAP patients, were included. The detailed sampling design is described in Materials and Methods. An overview is also available in Fig. 1. *Death status refers to patients who succumbed to the disease (patient with ID BCAP61 died from non-oncological reasons). Detailed clinicopathological information for BCAP and BCUS cohorts is provided in Additional File 2: Supplementary Table 1

### Whole-exome sequencing, data analysis, variant detection, and validation with independent methods

WES analyses were performed using the Agilent SureSelectXT Human All Exon V7 capture kit for sequencing library construction, followed by 150 bp paired-end sequencing on the HiSeq Illumina platform (Illumina, San Diego, CA), outsourced to Macrogen Europe (Amsterdam, The Netherlands). Sequencing coverage was 200x on average, with at least 100x on target. The sequencing coverage and quality statistics for each sample are summarized in Additional File 2: Supplementary Table 2 A.

FASTQ files were inspected and processed with *Trim Galore!* (v0.6.7) (https://www.bioinformatics.babraham.ac.uk/projects/trim_galore/) to remove Illumina-specific adapter sequences and poor-quality reads when necessary. After converting FASTQ files to BAM format and extracting read groups from the raw data, reads were processed using GATK4 Best Practices (v4.0) (https://github.com/gatk-workflows/seq-format-conversion, https://github.com/gatk-workflows/gatk4-data-processing). The reads were mapped to the human genome (hg38) using the BWA-MEM tool (http://bio-bwa.sourceforge.net*).* Octopus (v0.7.4), in cancer mode, was used for variant calling. The cancer calling model can jointly genotype multiple samples from the same individual, using a reference sample (whole peripheral blood or skin when blood was not available) to distinguish between post-zygotic and germline variants (https://luntergroup.github.io/octopus/*).* Frameshift insertions/deletions, nonsense, and missense variants located in exons were included in the analysis. A random forest filtering approach was implemented to minimize false calls. Variants in reads with poor mapping quality (< 30) and variants supported by high-quality bases (≥ 30) in fewer than five reads were excluded from the analysis. Variants were annotated using ANNOVAR (https://annovar.openbioinformatics.org/*)* (last updated on 07.07.2020 and accessed between 06.2022 and 08.2022) and wANNOVAR (https://wannovar.wglab.org/*)* (last accessed on 21.05.2024), using the MANE SELECT transcript for investigated genes (https://www.ensembl.org/info/genome/genebuild/mane.html). A brief overview of filtering strategies implemented for identifying post-zygotic and germline variants related to breast cancer, within BCAP, BCUS, and RM cohorts, and validation experiments for selected post-zygotic variants of BCAP patients are available in Additional File 1: Supplementary Fig. 2.

#### Post-zygotic variants

Only variants with sequencing depth ≥ 50 and tissue allele frequency ≥ 0.03 were included in the analysis. Variants were filtered based on their annotation in ClinVar and InterVar databases; variants reported as “pathogenic”, “likely pathogenic”, “uncertain significance”, or “conflicting interpretations of pathogenicity” were included. In parallel, variants described in the COSMIC database (Cosmic_95coding) were incorporated. Missense variants documented in the COSMIC database, but annotated as “benign” or “likely benign” in ClinVar or InterVar databases, were excluded. Variants in genomic regions with known read-through transcription between adjacent genes, or those spanning multiple genes (e.g., *P2RY11; PPAN-P2RY11* and *KIR2DL1; KIR2DS5; LOC112267881*), were also excluded.

The remaining variants were filtered by their frequency in the general population, retaining only those with a minor allele frequency (MAF) ≤ 0.001 across all gnomAD populations (“popmax”) or not listed in gnomAD (v2.1.1) (Additional File 2: Supplementary Table 3). Variants were further categorized as truncating (Additional File 2: Supplementary Table 4) or missense (Additional File 2: Supplementary Table 5). Truncating variants were considered pathogenic, regardless of their annotation in ClinVar or InterVar. For missense variants, in-silico analyses using the REVEL tool [18] (with a threshold score of 0.75) were performed. In summary, truncating variants, variants classified as “pathogenic” or “likely pathogenic” in ClinVar, and missense variants with conflicting interpretations or annotated as “pathogenic” in ClinVar without assertion criteria, but having a REVEL score ≥ 0.75, were deemed pathogenic.

To select post-zygotic variants potentially linked to breast cancer, we prioritized those annotated as “pathogenic” or “likely pathogenic” in the ClinVar database, reviewed by expert panels, or submitted by multiple parties without discordance (Additional File 2: Supplementary Table 6). Missense variants classified as of uncertain significance, pathogenic/likely pathogenic without assertion criteria, or with conflicting interpretations in ClinVar with a REVEL score ≥ 0.75 were also included. Truncating variants in known tumor-suppressor genes implicated in breast cancer, such as *KMT2C* [19], *TBX3* [20], and *TP53* [21], were included in the same table even if they were absent from the ClinVar database (Additional File 2: Supplementary Table 6). UM, UMD, PT, and SK samples from 8 BCAP patients were further investigated using Sanger sequencing or High-Resolution Melting (SS/ HRM) to verify the presence of selected variants (Table 2; Additional File 2: Supplementary Table 7; Additional File 1: Supplementary Fig. 3).

**Table 2:** Pathogenic post-zygotic variants within the uninvolved mammary tissue of BCAP patients, selected for further investigation/validation

Gene	Variant^a^	ClinVar^b^	COSMIC ID^c^	AVSNP150^d^	Individual ID and UM sample VAF^e^	Confirmation
*AKT1*	c.49G > A (p.Glu17Lys)	Pathogenic	ID=COSV62571334	rs121434592	BCAP32 (6%), BCAP66* (7%)	SS / HRM
*PIK3CA*	c.1624G > A (p.Glu542Lys)	Pathogenic	ID=COSV55873227	rs121913273	BCAP56 (7%), BCAP45 (12%)	SS / HRM
*PIK3CA*	c.3140 A > G (p.His1047Arg)	Pathogenic	ID=COSV55873195	rs121913279	BCAP15 (8%), BCAP31* (19%), BCAP36 (3%), BCAP53* (7%)	SS / HRM / DS
*PIK3CA*	c.3140 A > T (p.His1047Leu)	Pathogenic	ID=COSV55873401	rs121913279	BCAP54* (5%)	DS
*PTEN*	c.388 C > T (p.Arg130*)	Pathogenic	ID=COSV64288463	rs121909224	BCAP15 (7%)	SS / HRM
*TBX3*	c.371_372insTGGT (p.Ile125Profs*14)	n.a.	ID=COSV57471668	n.a.	BCAP44 (12%)	SS / HRM
*TP53*	c.151G > T (p.Glu51*)	Pathogenic	ID=COSV52694020	n.a.	BCAP58* (16%)	SS / HRM
*TP53*	c.227del (p.Ala76Aspfs*47)	n.a.	ID=COSV52728465	n.a.	BCAP54 (5%)	DS
*TP53*	c.329G > C (p.Arg110His)	Pathogenic	ID=COSV52668419	rs11540654	BCAP45 (8%)	DS
*TP53*	c.637 C > T (p.Arg213*)	Pathogenic	ID=COSV52665560	rs397516436	BCAP01* (8%), BCAP48* (6%)	DS
*TP53*	c.711G > A (p.Met237Ile)	Pathogenic	ID=COSV52661887	rs587782664	BCAP15 (8%)	SS / HRM
*TP53*	c.1024 C > T (p.Arg342*)	Pathogenic	ID=COSV52665487	rs730882029	BCAP38 (5%), BCAP47 (7%)	DS
*TP53*	c.1025G > C (p.Arg342Pro)	Pathogenic/Likely pathogenic	ID=COSV52690857	rs375338359	BCAP57 (6%)	DS

Presented variants, identified via Whole Exome sequencing in the uninvolved mammary (UM) samples of individuals characterized with adverse outcomes (Breast Cancer Adverse Prognoses cohort, BCAP), were corroborated with either Sanger sequencing/High-Resolution Melting, or Duplex sequencing. Detailed description of selected post-zygotic variants is provided in Additional File 2: Supplementary Table 6. Confirmation of post-zygotic variants by Sanger sequencing/High-Resolution Melting, or Duplex sequencing is provided in Additional File 1: Supplementary Figure and Additional File 2: Supplementary Tables 7 and 9, respectively

*SS* Sanger sequencing, *HRM* High-Resolution Melting, *DS* Duplex sequencing, *n.a. *not available

*variants were also detected in the distal uninvolved mammary gland sample (UMD) of selected patients

^a^Variant annotation provided for the basic isoform of the transcript

^b^Pathogenicity classification according to the ClinVar database

^c^ID of the variant in the COSMIC (Cosmic_95 coding) database

^d^rsIDs in dbSNP build 150

^e^Individual ID and Variant Allele Frequency (VAF) for UM samples

#### Germline variants

For germline variant detection, only variants in high- and moderate-penetrance breast cancer susceptibility genes were included in the study, as guided by the NCCN Clinical Practice Guidelines in Oncology [22] (Version 1.2023, September 7, 2022). The list of genes of interest includes: *ATM* (MIM *607585), *BRCA1* (MIM *113705), *BRCA2* (MIM *600185), *BARD1* (MIM *601593), *BRIP1* (MIM *605882), *CHEK2* (MIM *604373), *CDH1* (MIM *192090), *PALB2* (MIM *610355), *PTEN* (MIM *601728), *TP53* (MIM *191170), *NF1* (MIM *613113), *STK11* (MIM *602216), *RAD50* (MIM *604040), *RAD51C* (MIM *602774), *RAD51D* (MIM *602954) and additionally *PIK3CA* (MIM *171834). Variants were filtered based on their frequency in the general population: variants with minor allele frequency (MAF) ≤ 0.01 across all gnomAD populations (“popmax”) or not noted in the database (gnomAD v2.1.1) were included. Evidence according to the American College of Medical Genetics and Genomics and the Association for Molecular Pathology recommendations [23] was included to describe all germline pathogenic variants. Specifically, the evaluation of identified *BRCA1* and *BRCA2* variants was performed according to the Evidence-based Network for the Interpretation of Germline Mutation Alleles (ENIGMA) *BRCA1* and *BRCA2* Variant Curation Expert Panel [24] (Version 1.1.0) (Clinical Genome Resource, https://www.clinicalgenome.org/affiliation/50087/, https://cspec.genome.network/cspec/ui/svi/doc/GN092, https://cspec.genome.network/cspec/ui/svi/doc/GN097). Pathogenic germline variants meeting the study’s criteria, identified within breast cancer patients of the BCAP and BCUS cohorts, are described in Additional File 2: Supplementary Table 8.

### Duplex sequencing

UM samples from 11 BCAP patients were selected to investigate the presence of low-frequency *PIK3CA* and *TP53* variants beyond the detection limits of Sanger sequencing and High-Resolution Melting and a single, higher-frequency *TP53* variant, i.e., c.151G > T (p.Glu51*), located in a difficult GC-rich region. (Table 2; Additional File 2: Supplementary Table 9). Additionally, UMD samples, available for 6 of those patients, were included to explore further the distribution of selected variants in a more distant from the tumor, seemingly normal mammary tissue. Duplex sequencing was performed as previously described [15, 25].

### Duplex sequencing data analysis

Raw duplex sequencing data were analyzed using the Snakemake-based Duplex-seq-Pipeline (v1.1.4) (https://github.com/Kennedy-Lab-UW/Duplex-Seq-Pipeline*)* as previously described [26]. The sequencing coverage and quality statistics for each sample are summarized in Additional File 2: Supplementary Table 2B.

### Statistical analysis

All statistical analyses were carried out with in-house developed scripts using R Studio version 4.1.2 (2021-11-01). Packages *pheatmap* (version 1.0.12) and *ggplot2* (version 3.4.1) were used for plotting. Statistical significance of differences between two or multiple groups was tested using the Mann–Whitney U test or the Kruskal–Wallis H test, respectively. Statistical significance of features between multiple groups was tested with the Hypergeometric test or Fisher’s exact test. Hazard Ratios were calculated using the *coxph* function from the package *survival* (version 3.5-5). Kaplan-Meier analysis was performed using the *survfit* and *ggsurvplot* functions from the *survminer* package (version 0.4.9), and groups were tested with the log-rank test. Differences were considered significant at *p* < 0.05.

## Results

### Truncating post-zygotic variants in dosage-sensitive genes predominate in BCAP compared to BCUS and RM cohorts

We examined UM and PT sample sets from all breast cancer patients to identify variants associated with breast cancer (Fig. 1a). A BL or SK sample - used as a reference when blood was unavailable - helped distinguish post-zygotic from germline variants (Fig. 1b). Details of post-zygotic variants in the BCAP, BCUS, and RM cohorts that met the study’s cut-off criteria (Methods; Additional File 1: Supplementary Fig. 2) are summarized in Additional File 2: Supplementary Table 3. A significant age difference was observed between BCAP, BCUS, and RM cohorts (Kruskal-Wallis test, *p* = 7.7e-05), with the BCAP and BCUS cohorts being significantly older than the control group (Kruskal–Wallis H test, *p* = 0.000034 and *p* = 0.00036 for BCAP and BCUS, respectively). However, no significant difference was observed between the BCAP (median age: 62, range: 23–85) and BCUS (median age: 65, range: 37–84) cohorts (Kruskal–Wallis H test, *p* = 0.082).

Within the BCAP cohort, we identified 167 unique post-zygotic variants, corresponding to 174 total variant occurrences across 41 patients, indicating that several variants were shared by more than one individual. In contrast, the BCUS cohort harbored 56 variants in 24 patients, and the RM cohort carried 10 variants in seven individuals, all of which were either of uncertain significance or absent from ClinVar/InterVar. Across all three cohorts, the curated catalogue of coding post-zygotic variants comprised 240 events in total (Additional File 2: Supplementary Tables 3–5). In BCAP, these 174 events were distributed across 41 of 77 patients (53%), with a median of 3 variants per carrier (IQR 1–6; range 1–17; mean 4.24), and no single patient contributed more than 10% of all variants. In BCUS, 56 variants occurred in 24 of 49 patients (49%; median 1 variant, IQR 1-2.25; range 1–9; mean 2.33), while in the RM cohort 10 variants were found in 7 of 15 women (47%; median 1, IQR 1-1.5; range 1–3). Truncating variants in dosage-sensitive genes (39 events) - including unique nonsense (*n* = 25) and frameshift (*n* = 12) mutations, which lead to transcript elimination via nonsense-mediated mRNA decay [27] - were confined to 25 BCAP patients (Additional File 2: Supplementary Table 4), whereas non-truncating missense variants were present in all three cohorts. These per-individual distributions are summarized in Additional File 1: Supplementary Fig. 4A and 4B, respectively.

In the BCAP cohort, 29% (49/167) of the identified variants were deemed pathogenic. This list included truncating variants (*n* = 37), missense variants annotated as pathogenic/likely pathogenic (*n* = 8), and missense variants reported as uncertain significance or pathogenic/likely pathogenic in ClinVar, lacking assertion criteria but showing evidence of pathogenicity according to in-silico analyses (REVEL threshold set to 0.75 [18] ) (*n* = 4) (Additional File 2; Supplementary Tables 4 and 5). Notably, nearly one-quarter (24%, 12/49) of the pathogenic BCAP variants were detected only in the UM samples and were absent in the corresponding PTs. In comparison, the BCUS cohort had seven pathogenic variants, representing 13% of the total identified variants (*n* = 56). These included variants reported as pathogenic (*n* = 4), and missense variants classified as uncertain significance or pathogenic/likely pathogenic in ClinVar without assertion criteria, showing evidence of pathogenicity according to REVEL (*n* = 3) (Additional File 2: Supplementary Table 5). Nearly half (43%, 3/7) of the pathogenic BCUS variants were identified only in UM samples. The UM samples from the BCAP cohort were significantly enriched for pathogenic post-zygotic variants compared to those from the BCUS cohort (Hypergeometric test, *p* = 0.0008578).

We identified several variants affecting dosage-sensitive genes. These included deleterious variants in the tumor suppressors *KMT2C* [19], *PTEN* [27], *PTCH1* [28], *TBX3* [20], and *TP53* [21] as well as activating variants in oncogenes *AKT1* [29] and *PIK3CA* [30] identified in BCAP UM samples (Additional File 2: Supplementary Table 6). Oncogenes such as *SF3B1* [31], *HRAS* [32], and *GNAS* [33], and genes with a dual role in cancer (*RUNX1* [34]) were affected solely in the BCUS cohort, with only the latter two being dosage-sensitive (Additional File 2: Supplementary Table 6). *PIK3CA* was the only gene recurrently affected in UM samples from both BCAP and BCUS cohorts.

### Recurrence, coexistence, spatial distribution in the breast, and validation of variants

**Fig. 2:**
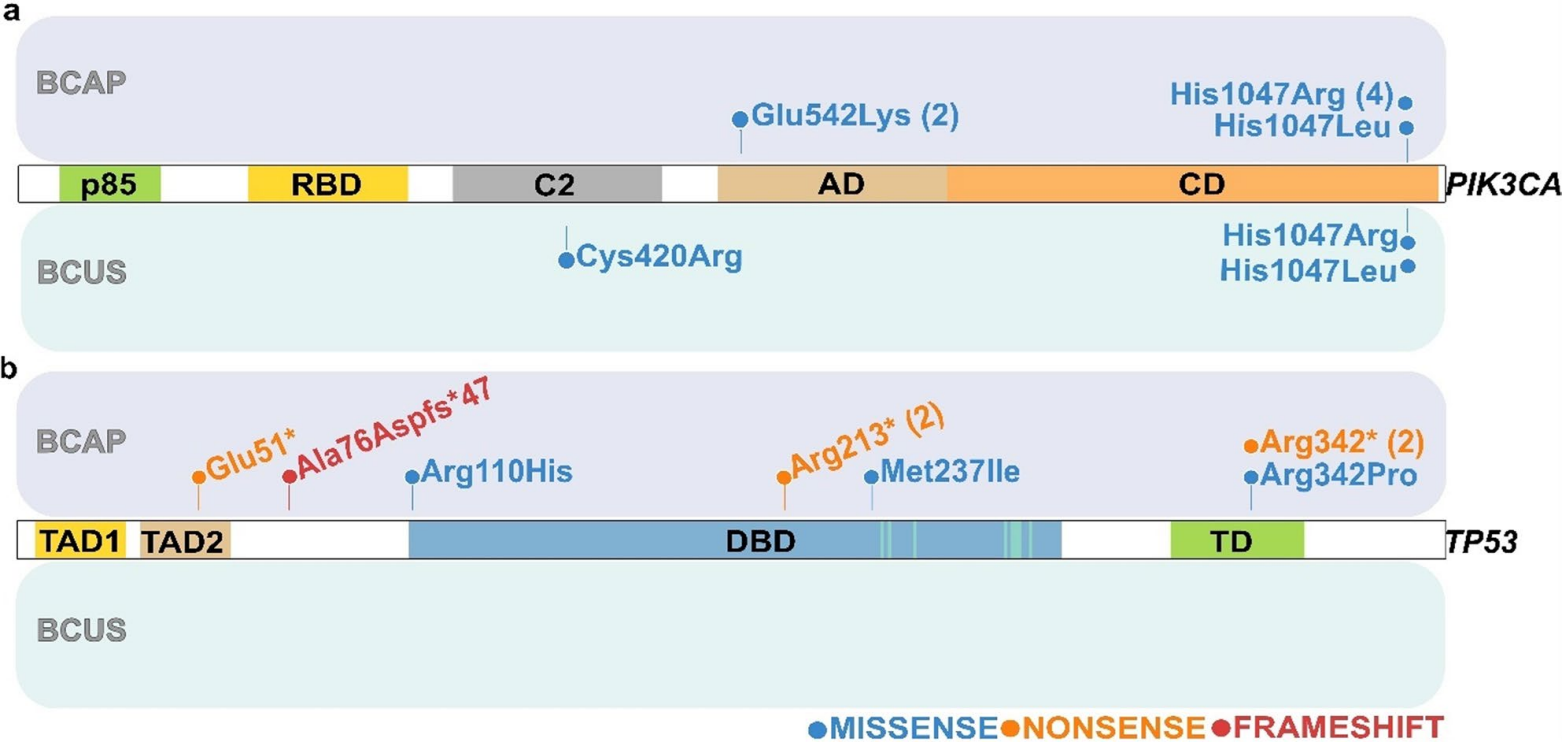
Post-zygotic (**a**) *PIK3CA* and (**b**) *TP53* variants detected in breast cancer patients. Pathogenic post-zygotic *PIK3CA* (**a**) and TP53 (**b**) variants were identified in the uninvolved mammary gland (UM) and/or primary tumor (PT) samples of breast cancer patients with adverse prognoses and of patients recruited without prognostic bias (BCAP and BCUS cohorts, respectively). Lollipop plots represent post-zygotic variants of *PIK3CA* and *TP53* detected by Whole-Exome Sequencing (WES). The upper panels display variants detected in UM samples from BCAP patients, and the lower panels those from BCUS patients. *TP53* variants were found exclusively in the BCAP cohort. Among the variants shown, three *PIK3CA* alterations, c.1624G > A (p.Glu542Lys) in BCAP45, c.3140 A > T (p.His1047Leu) in BCAP54, and c.1258T > C (p.Cys420Arg) in BCUS45, were detected only in UM samples and were absent from the corresponding PT tissues. All identified variants have been reported in the COSMIC database (https://cancer.sanger.ac.uk/cosmic). Detailed descriptions are provided in Additional File 2: Supplementary Table 6. p85 – p85-binding domain; RBD – Ras-binding domain; C2 – C2 domain; AD – accessory domain; CD – catalytic domain; TAD1, TAD2 – transcription activation domains 1 and 2; DBD – DNA-binding domain; DNA-binding sites are indicated in green; TD – tetramerization domain.Lollipop plots were prepared based on the images generated with the Protein paint application [36]. Numbers in parentheses indicate the number of patients in whom the variant was identified

In the BCAP cohort, pathogenic variants in two driver genes, *PIK3CA* and *TP53*, were predominant across all subtypes of invasive cancer. *PIK3CA*, which encodes the catalytically active p100alpha isoform, is a key regulator of cell proliferation and growth receptor signaling cascades [35]. We detected three distinct pathogenic post-zygotic *PIK3CA* variants in UM samples: c.1624G > A (p.Glu542Lys), c.3140 A > G (p.His1047Arg), and c.3140 A > T (p.His1047Leu), found in two, four, and one unrelated individuals, respectively (Additional File 2: Supplementary Table 5). Another *PIK3CA* variant, c.3012G > T (p.Met1004Ile), was found in the UM of a single BCAP cohort individual. However, this variant, reported only once as of uncertain significance in the ClinVar database and with a REVEL score of 0.437 suggesting it might be benign, was classified as a variant of uncertain significance due to limited evidence (Additional File 2: Supplementary Table 5). *PIK3CA* c.3140 A > G (p.His1047Arg) and c.3140 A > T (p.His1047Leu) variants, located at the commonly mutated *PIK3CA* site in breast cancer, were also observed in UMs of the BCUS cohort (Fig. 2; Additional File 2: Supplementary Table 5).

*TP53* is the most commonly mutated gene in various human cancers, and its normal protein function is frequently compromised in many types of malignancies [37]. We detected seven *TP53* variants in the normal mammary gland samples of nine BCAP patients, including two recurrent variants: six pathogenic or likely pathogenic (c.151G > T [p.Glu51*], c.329G > C [p.Arg110His], c.637 C > T [p.Arg213*], c.711G > A [p.Met237Ile], c.1024 C > T [p.Arg342*], and c.1025G > C [p.Arg342Pro]), and one frameshift variant c.227del (p.Ala76Aspfs*47), not previously reported in the ClinVar database (Additional File 2: Supplementary Table 6). Importantly, no *TP53* variants were observed in the normal mammary gland samples of the BCUS cohort (Fig. 2).

**Fig. 3:**
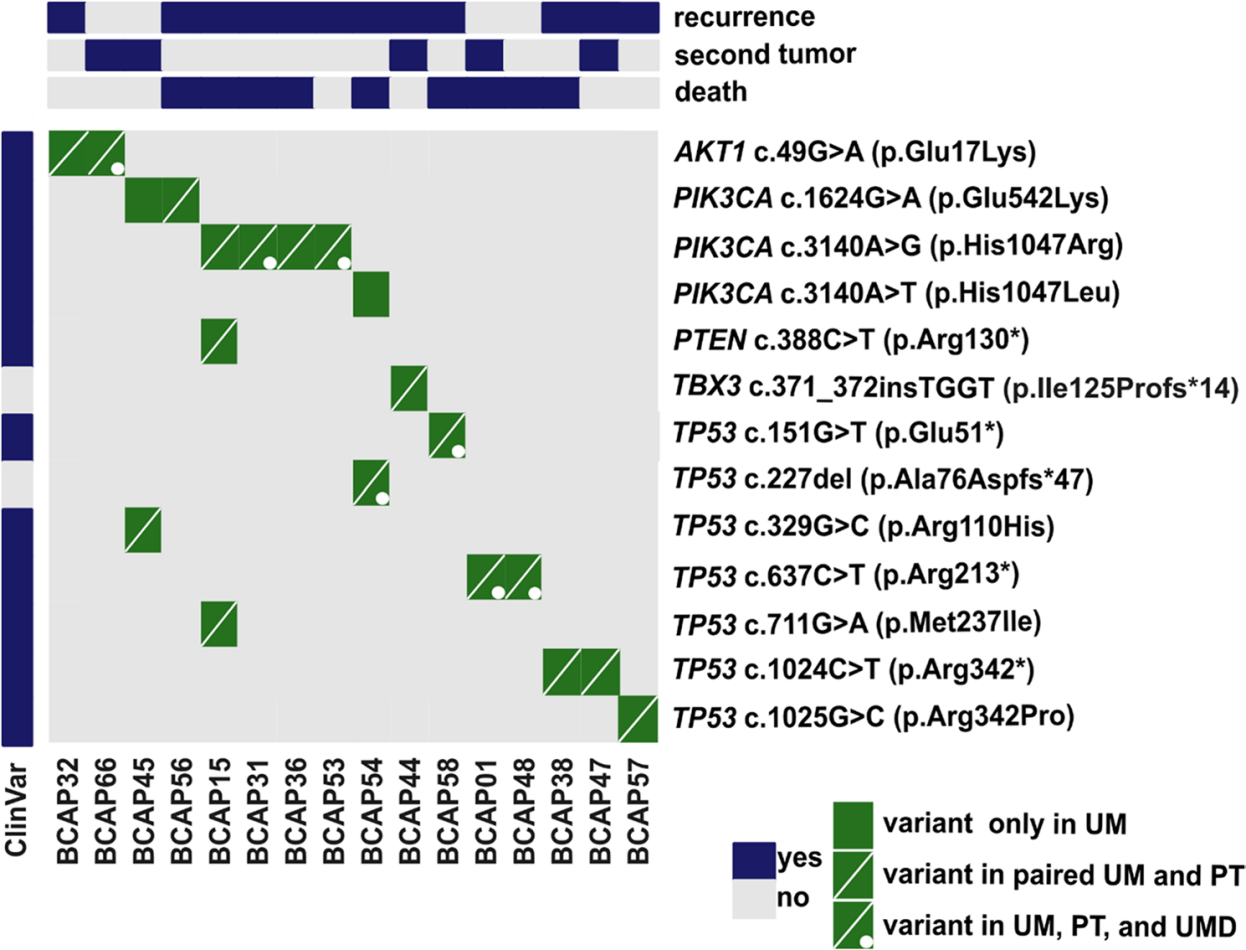
Pathogenic post-zygotic variants in breast cancer patients with adverse prognoses. Variants were confirmed via Sanger sequencing/High-Resolution Melting or Duplex sequencing (Methods; Table 2; Additional File 1: Supplementary Fig. 3; Additional File 2: Supplementary Tables 7 and 8). Variant presence in the ClinVar database and follow-up information for the corresponding patients are included. Detailed clinicopathological information for the presented patients is provided in Additional File 2: Supplementary Table 1. A full description of detected variants is provided in Additional File 2: Supplementary Table 6. PT – primary tumor. UMD – uninvolved mammary gland at a further distance from the corresponding PT

Pathogenic variants in *AKT1*, *PIK3CA*, *PTEN*, *TBX3*, and *TP53* in 16 BCAP patients were subsequently selected for orthogonal validation using Sanger sequencing/High-Resolution Melting and/or duplex sequencing (Table 2). In total, 13 pathogenic post-zygotic variants were tested, and all 13 were confirmed. Seven variants were validated by SS/HRM in UM and/or UMD samples, and seven by duplex sequencing, with the *PIK3CA* hotspot variant p.His1047Arg confirmed by both approaches. These analyses also showed that in several patients the same pathogenic variant was detectable in both proximal and distal uninvolved mammary gland tissue, indicating a broad spatial distribution of mutant clones in the breast. Of particular note, patient BCAP15 carried concurrent pathogenic variants in *PIK3CA*, *TP53*, and *PTEN* in UM tissue, exemplifying the co-occurrence of multiple driver events in histologically normal mammary epithelium (Fig. 3; Supplementary Figs. 2 and 3; Supplementary Tables 6–9).

To extend these analyses to variants close to the detection limit of WES and SS/HRM, we performed ultra-deep duplex sequencing in UM and, when available, UMD samples from 11 BCAP patients with *PIK3CA* or *TP53* variants (Table 2). At a mean duplex coverage of 4,789×, this approach confirmed all low-frequency (as low as 1.34%) *PIK3CA* and *TP53* variants identified by WES and demonstrated their presence in both proximal and distal uninvolved tissue in a subset of cases, further supporting the existence of spatially extended mutant fields in the breast (Table 2; Fig. 3, Supplementary Table 9).

### Spectrum of germline pathogenic variants in the two breast cancer cohorts

All breast cancer cases included in our study were reported as sporadic, and in most instances germline genetic testing results were not available prior to recruitment. We therefore analysed germline DNA from BL or SK samples of all participants to screen for pathogenic and likely pathogenic variants in established breast cancer predisposition genes (Additional File 1: Supplementary Fig. 2). In the BCAP cohort, 14 of 77 individuals (18%) carried pathogenic germline variants in these genes, including 8 with variants in *BRCA1*, 3 in *BRCA2*, 3 in *PALB2* and 1 in *RAD50*. In the BCUS cohort, 1 of 49 individuals (2%) carried a pathogenic *BRCA1* variant. The most frequent alteration was the *BRCA1* founder variant c.5266dup (p.Gln1756Profs*74), observed in four BCAP cases and one BCUS case. This proportion of carriers is higher than typically reported in unselected breast cancer series and likely reflects the enrichment of the BCAP cohort for patients with adverse clinical outcomes [15, 21, 38]. Notably, four BCAP patients with germline pathogenic variants also harboured pathogenic post-zygotic variants in curated cancer-related genes in their UM samples, illustrating that inherited predisposition can coexist with somatic mosaicism in histologically normal breast epithelium. Full details of individual germline variants are provided in Additional File 2: Supplementary Table 8.

### Intrinsic subtype differences do not account for UM post-zygotic variant patterns

To determine whether clinical or molecular subtype differences between cohorts could account for the distribution of post-zygotic variants in uninvolved mammary tissue, we compared receptor status and intrinsic subtype between BCAP and BCUS and formally tested associations with UM variant detection. As shown in Table 1, the cohorts differed significantly in Estrogen (ER) and Progesterone (PR) status (ER: 74.0% vs. 87.8%, *p* = 0.040; PR: 55.8% vs. 89.8%, *p* = 1.7 × 10⁻⁵) and in intrinsic subtype composition (χ² (3) = 14.3, *p* = 0.0025), with BCUS enriched for Luminal A tumours and BCAP containing more Luminal B and Triple-Negative Breast Cancer (TNBC) cases. However, detection of coding post-zygotic variants in UM did not differ by ER status (52.0% vs. 52.0%; *p* = 1.00), HER2 status (52.4% vs. 51.5%; *p* = 1.00), or intrinsic subtype (HER2-enriched 63.6%, Luminal A 41.7%, Luminal B 56.9%, TNBC 33.3%; χ² (3) = 0.24). Likewise, the per-individual UM variant burden was comparable across subtypes (Kruskal-Wallis *p* = 0.25). These analyses indicate that the observed post-zygotic variant patterns in UM are not explained by underlying differences in receptor status or intrinsic subtype distribution between cohorts.

To further assess whether the intrinsic subtype might influence the number of variants carried by each individual, we examined per-patient post-zygotic variant burden stratified by subtype. Across the BCAP and BCUS cohorts, 65 patients carried at least one coding post-zygotic variant; however, intrinsic subtype information was available for 59 of them, with the remaining six lacking complete receptor or subtype annotation (Supplementary Tables 3 and 1, respectively). These 59 individuals formed the denominator for the subtype-stratified burden analyses: HER2-enriched (*n* = 7), Luminal A (*n* = 15), Luminal B (*n* = 33), and TNBC (*n* = 4). Consistent with the presence/absence analyses above, total coding post-zygotic variant burden did not differ significantly across intrinsic subtypes (Kruskal-Wallis H = 3.14, *p* = 0.37), and no pairwise Mann-Whitney comparison reached significance (*p* ≥ 0.20) (Additional File 1, Supplementary Fig. 4C). Analyses of pathogenic post-zygotic variants were further restricted to the 24 individuals carrying at least one pathogenic variant in curated cancer-related genes. Pathogenic variant burden was likewise similar across subtypes (HER2 *n* = 5; Luminal A *n* = 5; Luminal B *n* = 11; TNBC *n* = 3), with no statistically significant differences observed (Kruskal–Wallis H = 5.89, *p* = 0.12; all pairwise *p* ≥ 0.06), although TNBC cases showed a modest upward trend (Additional File 1: Supplementary Fig. 4D). Together, these findings indicate that neither subtype composition nor subtype-specific variation in individual variant burden accounts for the higher UM post-zygotic variant burden observed in BCAP.

## Discussion

The early detection and treatment of breast cancer, including its precursors, have shifted research focus from tumors to the normal mammary gland to deepen our understanding of the disease’s origins [39]. Genetic and transcriptomic studies have revealed a wide spectrum of alterations in critical breast cancer driver genes within the normal mammary gland of patients who have undergone BCS or mastectomy, compared to control tissues [11–15, 40].

Recognizing that histologically normal tissue may harbor early genetic changes, we screened for post-zygotic alterations in two similarly aged breast cancer cohorts (BCAP and BCUS, Kruskal–Wallis H test, *p* = 0.082) with differing survival outcomes. We also included a significantly younger control group of individuals treated surgically for non-cancerous reasons (RM cohort, Kruskal–Wallis H test, *p* = 0.000034 and *p* = 0.00036 for BCAP and BCUS cohorts, respectively).

Truncating variants (nonsense and frameshift) were found exclusively in the UM samples of patients with adverse prognoses (BCAP cohort) (Additional File 2: Supplementary Table 4). In contrast, BCUS patients and RM controls showed only missense variants (Additional File 2: Supplementary Table 5). UM samples from BCAP patients were significantly enriched for pathogenic post-zygotic variants (Hypergeometric test, *p* = 0.0008578), affecting several known cancer-related genes [41], i.e., *AKT1*, *KMT2C*, *PIK3CA*, *PTCH1*, *PTEN*, *TBX3*, and *TP53*, and almost a quarter were found exclusively in UM samples, absent from the corresponding PTs, suggesting genetically distinct clonal events in morphologically normal tissue.

Post-zygotic variants in canonical driver genes *PIK3CA* and *TP53* were markedly enriched in UM tissue from BCAP patients and largely absent from UM in the unselected BCUS cohort (Fig. 2; Additional File 2: Supplementary Tables 5 and 6). In several BCAP cases, *PIK3CA* and *TP53* variants co-occurred in UM, and in one case alongside a pathogenic *PTEN* variant, consistent with genetically advanced post-zygotic fields rather than isolated lesions. The concentration of such complex mutant fields in BCAP, but not BCUS at the time of surgery, suggests that clonally expanded post-zygotic alterations contribute to the biological heterogeneity of breast tissue. The presence of alterations affecting multiple major cancer genes within single individuals highlights that genetic mosaicism is a feature of apparently normal mammary tissue and underscores the importance of comprehensive molecular profiling when studying disease biology. Importantly, although the BCAP and BCUS cohorts differed significantly in intrinsic subtype composition, neither the presence nor the burden of post-zygotic variants in the uninvolved mammary gland was associated with subtype. This indicates that the enrichment of pathogenic post-zygotic variants in BCAP cannot be attributed to subtype differences and instead reflects genuine underlying differences in clonal architecture between cohorts.

While post-zygotic variants in *TP53* or *PIK3CA* have been observed in breast tumors, their consequences in normal mammary tissue are less clear. Some studies suggest a benign effect [42, 43]. Healthy breast tissue accumulates alterations with age at an accelerating rate [44] and is influenced by hormonal stimuli, undergoing cycles of expansion during puberty, pregnancy, and lactation [45]. Estrogen and its metabolites can cause DNA damage, increasing cellular stress and the risk of genetic alterations and cancer [45]. However, the accumulation of alterations alone does not cause cancer; observed tissue-specific patterns and the “ground state” theory suggest that quiescent stem cells with oncogenic variants rarely transform unless activated by developmental, aging, or injury factors, which normally resemble physiological mammary gland conditions [46]. The presence of such variants in ostensibly normal mammary gland tissue suggests they may represent early, pre-cancerous changes. Despite the mammary gland’s inherent multi-layer protection system against clonal expansions, surviving mutant clones can lead to large fields of mutated cells, i.e., field cancerization, thereby increasing cancer risk [47]. Notably, the BCAP and BCUS cohorts were age-matched, indicating that the striking differences in UM variant burden between them are unlikely to be driven by age-related accumulation alone.

A subset of the BCAP cohort (18%, *n* = 14/77) and one patient from the BCUS cohort (2%, *n* = 1/49) carried germline pathogenic variants in breast cancer genes (Additional File 2: Supplementary Table 8). Among the BCAP patients with these variants, four had concurrent pathogenic post-zygotic variants in curated cancer genes, whereas 14 had only post-zygotic alterations in curated cancer genes and genes implicated in breast cancer (Additional File 2: Supplementary Tables 6 and 8). Despite their differing genetic profiles, all BCAP patients experienced adverse outcomes within ten years of surgery, highlighting the impact of these genetic variations on prognosis. The interaction between germline and post-zygotic variants remains unclear, as recent research indicates that the influence of germline variants on tumor behavior can vary significantly based on factors such as penetrance and lineage, with some variants exhibiting minimal or transient effects on tumor development [48]. Notably, the uniformly poor outcomes among BCAP patients occurred irrespective of whether risk was driven by germline predisposition, post-zygotic mosaicism, or both, suggesting that expanded post-zygotic fields may exert prognostic influence independent of inherited susceptibility.

Current diagnostics primarily focus on identifying germline pathogenic variants in known breast cancer-associated genes to assess breast cancer risk and guide personalized therapy [22]. However, over 80% of breast tumors are not caused by inherited alterations [9]. Our study reveals that pathogenic post-zygotic variants, such as *PIK3CA* and *TP53* alterations, are often found in seemingly normal mammary gland tissue left behind after BCS, with allele frequencies ranging from 0.03 to 0.28. In some instances, these variants represent distinct clonal populations not present in the corresponding primary tumors (Additional File 2: Supplementary Table 6), indicating the independent evolution of cell lineages within the mammary tissue and suggesting they are unlikely to be micrometastases.

However, although the detection of variants not shared with the corresponding primary tumors supports the presence of genetically distinct clonal populations, the possibility of occult tumor cells escaping histopathological detection cannot be excluded. Both pre-existing mutant fields and undetected tumor cell dissemination could contribute to recurrence risk, yet remain difficult to distinguish without longitudinal clonal tracking. The absence of certain truncal tumor mutations in uninvolved mammary tissue, despite the presence of other variants, may support early clonal divergence and field formation, whereas fully overlapping mutational profiles would favor occult tumor spread. As recurrence samples were not available, this distinction cannot be resolved and should be interpreted with caution. Future studies integrating matched primary, adjacent, and recurrence tissues will be essential to define clonal relationships and clarify the role of post-zygotic mosaicism. In line with this uncertainty, the timing of emergence of these variants relative to the primary tumor, whether during early tumor progression or later, remains uncertain, but in both scenarios, they may contribute to recurrence in the breast or metastasis to other organs [49]. Understanding these dynamics is essential for deciphering how post-zygotic mosaicism contributes to mammary gland biology.

Our study comes with certain limitations, particularly regarding the notable age differences between the breast cancer cohorts and individuals subjected to reduction mammoplasty surgeries. Recruiting age-matched control individuals poses a challenge, as those opting for cosmetic surgical treatments are typically younger. The incidence of breast cancer diagnosis among younger women is relatively infrequent, with only about one out of eight invasive breast cancers being diagnosed in women under the age of 45 [1]. Another limitation arises in recruiting healthy control individuals, given that approximately 13% of women are expected to develop invasive breast cancer during their lifetime and the precise onset of carcinogenesis remains unclear [1, 2]. Here, control normal mammary glands were sampled from individuals without a personal or familial history of cancer undergoing plastic surgery.

Overall, our findings show that pathogenic post-zygotic variants in cancer-associated genes are more prevalent in histologically normal mammary tissue from BCAP patients compared with BCUS or RM individuals. The presence of such variants in morphologically normal tissue raises important questions about early clonal dynamics in the breast and highlights potential blind spots in tumor-centric views of breast cancer biology. Further work is needed to determine how these mosaic events emerge, persist, and potentially contribute to future disease development.

## Conclusions

Our study reveals that histologically normal mammary tissue from breast cancer patients, particularly those with poor prognoses, frequently harbors pathogenic post-zygotic variants in key cancer-associated genes such as *PIK3CA* and *TP53*. These alterations are often distinct from those found in the primary tumor, suggesting independent clonal evolution and potential early oncogenic activity. These findings highlight the value of characterizing post-zygotic variants not only within tumors but also in adjacent morphologically normal tissue. Incorporating such analyses into breast cancer research and, in the future, potentially into clinical workflows may refine risk stratification and improve our understanding of early clonal dynamics in the mammary gland.

## Supplementary Information

Supplementary Material 1.

Supplementary Material 2.

## Data Availability

Raw duplex sequencing and WES data are available upon request in the EGA archive, under study IDs EGAS50000000538 and EGAS50000000539, respectively.

## Abbreviations

ER: Estrogen
BCAP: Breast Cancer Adverse Prognoses
BCS: Breast-conserving surgery
BCUS: Breast Cancer Un-Selected
BL: Whole Peripheral Blood
HRM: High Resolution Melting
PR: Progesterone
PT: Primary Tumor
RM: Reduction Mammoplasty
SK: Skin
SS: Sanger sequencing
TNBC: Triple-Negative Breast Cancer
UM: Uninvolved mammary gland
UMD: Distal uninvolved mammary gland
WES: Whole Exome Sequencing
